# Trends in Anti‐Müllerian Hormone (AMH) Testing for Fertility Assessment in Australian General Practice, 2011–2021: A Retrospective Open Cohort Study

**DOI:** 10.1111/ajo.70179

**Published:** 2026-08-31

**Authors:** Gizat M. Kassie, Tessa Copp, Sarah Lensen, Danielle Mazza, Jacqueline A. Boyle, Luke E. Grzeskowiak

**Affiliations:** ^1^ College of Medicine and Public Health Flinders University Adelaide South Australia Australia; ^2^ SAHMRI Women and Kids Theme South Australian Health and Medical Research Institute Adelaide South Australia Australia; ^3^ Sydney Health Literacy Lab, Faculty of Medicine and Health, School of Public Health The University of Sydney Sydney Australia; ^4^ Department of Obstetrics, Gynaecology and Newborn Health The University of Melbourne Parkville Victoria Australia; ^5^ Department of General Practice, School of Public and Preventive Medicine Monash University Melbourne Victoria Australia; ^6^ Department of Health Systems and Equity, Eastern Health Clinical School Monash University Melbourne Victoria Australia

**Keywords:** AMH, anti‐mullerian hormone, fertility, reproductive health

## Abstract

**Background:**

Anti‐Müllerian hormone (AMH) tests are often promoted as a measure of fertility; however, AMH adds limited predictive value beyond age alone for estimating the likelihood of live birth in couples trying to conceive.

**Aims:**

This study aimed to examine longitudinal trends in the rate of AMH testing for fertility in the Australian general practice setting.

**Materials and Methods:**

Retrospective open cohort study of 1,393,931 females aged 20–44 years attending Australian general practices between 2011 and 2021 using the national general practice database, MedicineInsight. Free text ‘test reason’ fields were examined to identify AMH tests ordered by General Practitioners for fertility assessment. We calculated age‐standardised annual prevalence rates for AMH tests overall, as well as stratified by age group and concession card status. To examine between‐practice variation, the intraclass correlation coefficient (ICC) was estimated using a two‐level logistic regression model.

**Results:**

The annual age‐standardised prevalence of AMH test requests increased from 0.32 per 1000 females in 2011 to 4.33 per 1000 females in 2021. AMH testing was more common among those residing in a major city and of higher socioeconomic status, but less common among concession card holders. Following adjustment for patient case‐mix, 21% (95% CI 18%–24%) of observed variation in AMH testing was attributable to differences between practices, with 45/423 (10.6%) of general practices having no patients with an AMH test.

**Conclusions:**

The noted substantial increase in clinical and consumer demand for AMH testing does not appear to be evidence‐based, with substantial patient‐ and practice‐level variation in testing evident.

## 1 Background

Anti‐Mullerian hormone (AMH) is produced by the granulosa cells surrounding each oocyte in the developing ovarian follicle and serum AMH levels are reflective of a woman's ovarian reserve, naturally declining as women age [1]. AMH levels are established as a strong predictor of ovarian response to exogenous gonadotrophin stimulation and provide an indication of the likely number of eggs that can be retrieved in a stimulated cycle for in vitro fertilisation (IVF) or egg freezing [2]. This can prove useful in setting expectations for patients while also facilitating tailoring of gonadotrophin doses to reduce the risk of ovarian hyperstimulation syndrome [3].

Because AMH reflects ovarian reserve, it has often been interpreted more broadly as a potential age‐independent marker of reproductive capacity and has therefore been promoted as a ‘fertility test’. However, research has consistently demonstrated that AMH adds limited predictive value beyond age alone for estimating the likelihood of live birth, in both assisted reproductive technology and spontaneous conception settings [4, 5, 6]. AMH reflects oocyte quantity rather than oocyte quality, and low AMH levels do not necessarily indicate a reduced chance of pregnancy among women trying to conceive naturally [3]. Accordingly, AMH testing is not recommended by major guidelines for women who are curious about their current or future fertility, or who are attempting conception without evidence of infertility [3, 7, 8, 9, 10, 11]. Further, guidelines indicate that in women with regular cycles, ovarian reserve testing is not required to diagnose unexplained infertility and is not predictive of spontaneous conception [7]. Collectively, evidence suggests there is limited value for AMH tests conducted for assessing fertility outside of specialist settings; however, international and national data on the uptake of AMH testing for assessing fertility in the primary care setting remains limited. In a 2022 Australian cross‐sectional survey of 1773 females aged 18–55 years, 7% reported having had an AMH test, with approximately 1 in 3 (31%) accessing the test through their General Practitioner (GP) [12]. This survey also revealed that 35% reported testing for elective reasons not supported by current evidence, including wanting to understand their chances of conceiving (19%), curiosity about fertility (9%), considering egg freezing (5%), and considering delaying pregnancy (2%) [12]. In view of scarce data about the use of this low‐value test we aimed to examine longitudinal trends in AMH testing for fertility assessment among almost 1.4 million reproductive aged females attending Australian general practice.

## 2 Materials and Methods

### 2.1 Study Design and Data Source

This was a retrospective cohort study using data from a nationally representative general practice research dataset, MedicineInsight, from 1 January 2011 to 31 December 2021. The MedicineInsight dataset has been described in detail elsewhere [13]. The MedicineInsight dataset collects data on individual demographics, practice encounters (not including progress notes), diagnoses, and pathology tests. MedicineInsight contains electronic health records (EHRs) from > 2000 GPs and > 400 general practices across Australia (8.2% of all Australian practices) [13]. The characteristics of MedicineInsight patients have been previously demonstrated to be nationally representative [14].

The independent MedicineInsight Data Governance Committee approved the study (protocol 2019–003), and the Human Research Ethics Committee of the University of Adelaide exempted it from ethical review due to the use of non‐identifiable data. We adhered to the guidelines outlined in the REporting of studies Conducted using Observational Routinely‐collected Data (RECORD) statement while reporting this study [15].

### 2.2 Study Population

The study cohort consisted of females aged 20–44 years attending general practice between 1 January 2011 to 31 December 2021 who were considered active patients (attended the same general practice three or more times). We excluded those with a documented diagnosis of poly‐endocrine metabolic ovarian syndrome (PMOS; recently renamed from polycystic ovarian syndrome [PCOS]) [16], given clinical practice guideline recommendations that AMH testing may be of value in the diagnosis of PMOS [17].

### 2.3 AMH Testing

We limited our analysis to AMH tests requested by GPs. AMH tests were identified by searching the free text requested test and result name fields for “AMH” or ‘Anti‐Müllerian hormone’ (including related spellings). Reasons for AMH testing were identified by searching the free text “test reason” field for terms related to fertility/infertility (e.g., ‘egg timer test’, ‘check fertility’, ‘subfertility’). We excluded AMH tests where the test reason indicated that it was for assessing PMOS, or where no test reason was provided or indicated an alternative test reason (e.g., menopause). Further, we excluded test reasons indicating the likely management (rather than assessment) of infertility (e.g., IVF, ICSI) or being related to ovarian stimulation procedures (e.g., egg donation, cryopreservation). Detailed descriptions of free text search terms used to determine the test reason are provided in Table S1.

Where available we extracted the AMH test value, the documented reference range (whether a fixed range irrespective of age, or variable range based on age), and whether or not the test result was flagged as being ‘abnormal’ or ‘low’ using the reference ranges documented by the pathology provider.

### 2.4 Covariates

Patient characteristics at the time of the first requested AMH test were extracted and included age, remoteness and socio‐economic indexes for areas (SEIFA), state/territory, concession card status and smoking status. Remoteness, socio‐economic indexes for areas (SEIFA) and State/territory were based on patients' residential postcodes. Remoteness was determined in accordance with the Australian Bureau of Statistics' (ABS) Australian Statistical Geography Standard (ASGS) remoteness areas, with 1 being a major city and 5 a very remote area. Due to small population sizes, data for ‘Remote’ and ‘Very Remote’ were combined. SEIFA was determined according to the ABS Index of Relative Socio‐Economic Advantage and Disadvantage (IRSAD) codes.

### 2.5 Statistical Analysis

Descriptive statistics (counts and percentages) were used to describe the study population. Prevalence rates were calculated based on those who received at least one AMH test in a given calendar year and were expressed per 1000 females. Prevalence rates were further stratified by age category (20–24, 25–29, 30–34, 35–39, 40–44). Prevalence rates (except for those stratified by age) were directly age‐standardised against the 2001 Australian standard population [18]. We used univariable generalised linear regression models with Poisson distribution and log link function and cluster variable according to practitioner ID, to compare the likelihood of AMH testing, generating relative risks (RR) and 95% confidence intervals (CI). We applied the same models for comparing the likelihood of having two or more requested AMH tests. In order to examine variability in the prevalence of AMH testing for fertility reasons across general practices, we calculated the intraclass correlation coefficient (ICC), estimated using an empty two‐level logistic regression model and a two‐level logistic regression model adjusted with patient variables including age, socioeconomic status, and concessional status (to reflect differences in patient case‐mix between general practices). The ICC represents an estimation of the proportion of the total variation that is attributable to the variation between general practices [19]. The statistical analysis was performed using STATA MP 18 (Stata, College Station, Texas) with graphs prepared using R (R Core Team, 2022).

### 2.6 Patient and Public Involvement

Patients and/or the public were not involved in the design, conduct, reporting or dissemination plans of this research.

## 3 Results

MedicineInsight data were available for 1,393,931 females aged 20–44 years who were active general practice patients during 2011–2021. A total of 12 060 females had at least one AMH test requested by their GP, of which 8621 had a documented test reason related to fertility assessment (Figure S1).

**FIGURE 1:**
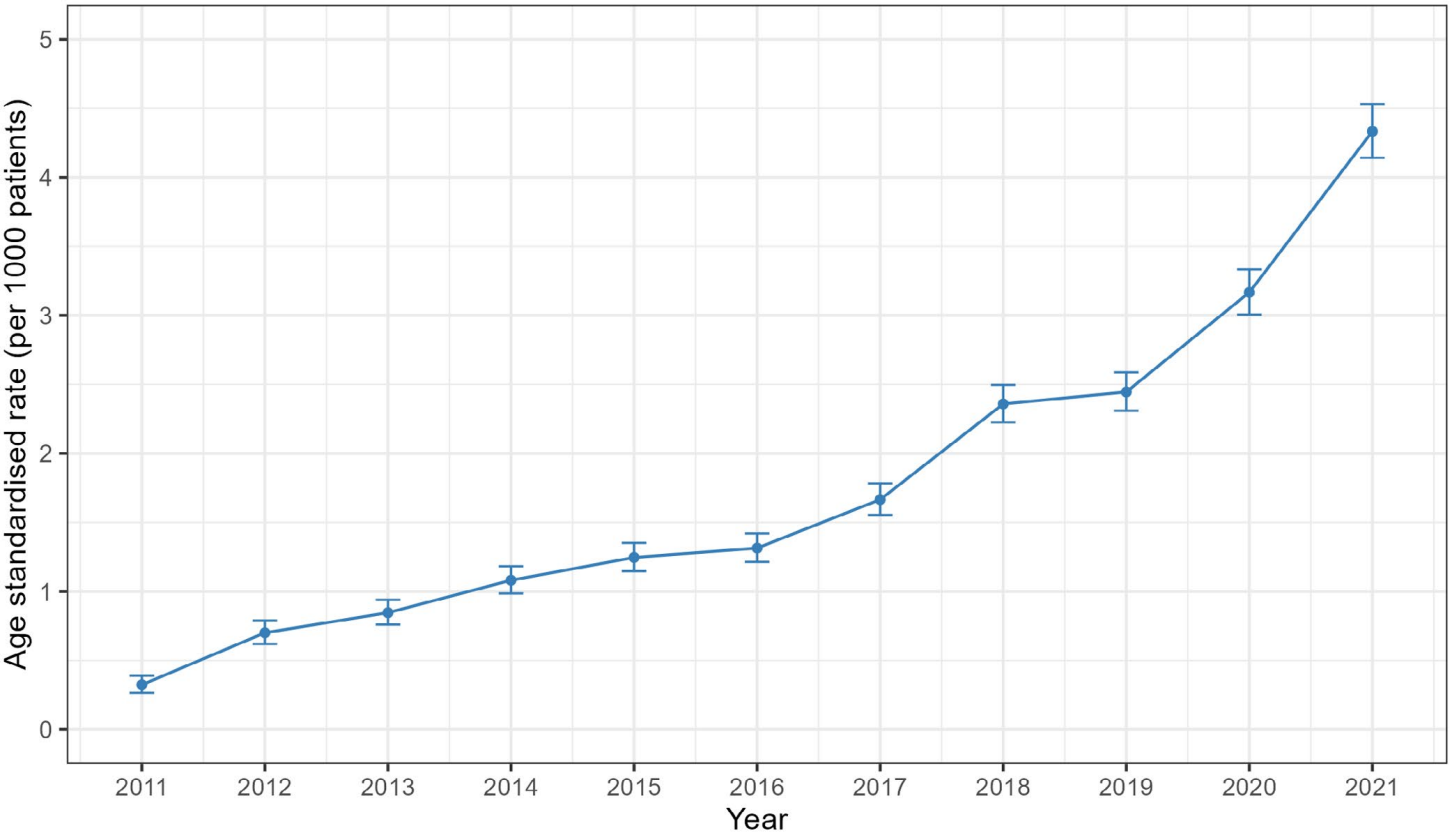
Age standardised annual prevalence rate of Anti‐Mullerian hormone (AMH) tests requested for fertility assessment among females aged 20–44 years attending Australian general practice, 2011–2021.

The annual age‐standardised prevalence of AMH test requests for fertility increased from 0.32 per 1000 females in 2011 to 4.33 per 1000 females in 2021 (Figure 1).

**FIGURE 2:**
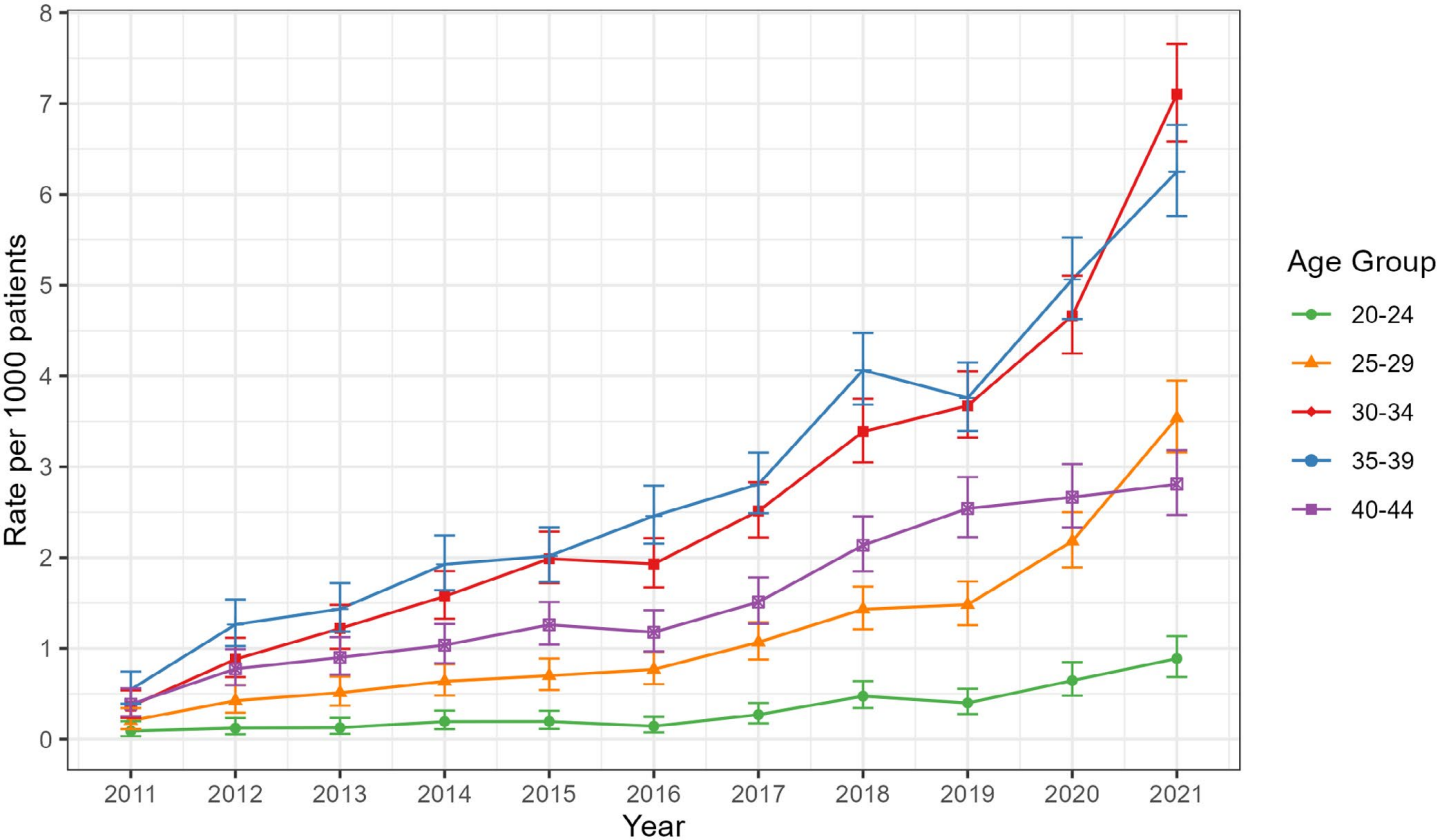
Annual prevalence rate of Anti‐Mullerian hormone (AMH) tests requested for fertility assessment among females aged 20–44 years attending Australian general practice stratified by age group, 2011–2021.

The median age at the time of first AMH test request for fertility reasons lowered slightly from 36 years (IQR 32–39 years) in 2011 to 34 years (IQR 30–37 years) in 2021. Prevalence of AMH tests over time for fertility reasons increased by 7‐ to 19‐fold among all age groups, with highest absolute and relative rate increase observed for those aged 30–34 years, increasing from 0.36 per 1000 females in 2011 to 7.10 per 1000 females in 2021 (Figure 2).

Characteristics associated with an increased likelihood of receiving an AMH test for fertility reasons included residing in a major city, higher socioeconomic status, being a non‐smoker and not having a concession card (Table 1).

**TABLE 1:** Characteristics of females aged 20–44 years who attended Australian general practice according to whether they had an AMH test requested for fertility assessment, 2011–2021.

Variable	No AMH test requested	AMH test requested	RR (95% CI)
	1 339 838	8621	
Socioeconomic status			
Very low	194 752 (14.6%)	658 (7.7%)	0.37 (0.34–0.41)
Low	231 535 (17.4%)	1140 (13.3%)	0.54 (0.51–0.58)
Middle	266 594 (20.0%)	1549 (18.1%)	0.64 (0.60–0.68)
High	289 373 (21.8%)	2071 (24.1%)	0.79 (0.75–0.83)
Very high	348 188 (26.2%)	3160 (36.8%)	Reference
Patient remoteness			
Major city	889 721 (66.9%)	6573 (76.6%)	Reference
Inner regional	286 338 (21.5%)	1381 (16.1%)	0.65 (0.62–0.69)
Outer regional	133 789 (10.1%)	513 (6.0%)	0.52 (0.48–0.57)
Remote & Very remote	20 453 (1.5%)	111 (1.3%)	0.74 (0.61–0.89)
Health care concession card status			
No concession	1 071 414 (80.0%)	7832 (90.8%)	Reference
Concession	268 424 (20.0%)	789 (9.2%)	0.40 (0.38–0.43)
Smoking status			
Non‐smoker	561 760 (50.1%)	4511 (57.5%)	Reference
Ex‐smoker	438 167 (39.1%)	2883 (36.7%)	0.82 (0.78–0.86)
Smoker	121 586 (10.8%)	451 (5.7%)	0.46 (0.42–0.51)

*Note:* Percentages were calculated out of the total number of patients in the respective columns.

Abbreviations: AMH, Anti‐Mullerian Hormone; CI, confidence interval; RR, relative risk.

Among the 8621 requested AMH tests, 5659 (65.6%) had a documented value and flag present indicating whether or not the value was considered ‘low’. A total of 1089/5659 (19.2%) levels were flagged as being ‘low’. Reference ranges were documented for 4561/5659 (80.6%) of AMH tests, with 1602/4561 (35.1%) utilising a fixed reference range irrespective of age at testing and 2959/4561 (64.9%) utilising a variable reference range according to age at testing. The proportion of AMH tests flagged as being ‘low’ was significantly higher among those where a fixed reference range was utilised (790/1602; 49.3%) compared with a variable reference range (299/2959; 10.1%).

The proportion of patients receiving an AMH test for fertility varied across practices from 0% to 3.77% (IQR 0.18% to 0.79%). When measured using the ICC, 21% (95% CI 18%–24%) of the variation in AMH testing was attributable to differences between practices, rather than patient factors. A total of 45/423 (10.6%) of general practices had no patients with a requested AMH test.

A total of 336 (3.9%) females had two or more documented AMH tests. No factors were significantly associated with an increased likelihood of repeat AMH testing (Table S2). The median time between repeated tests was 180 days (IQR 76–516). Among the 93 who were flagged as being “low” on the first test, 32 (34%) were flagged as being “normal” on the second test.

## 4 Discussion

### 4.1 Main Findings

The prevalence rate of AMH testing for assessing fertility among females of reproductive‐age increased more than 13‐fold between 2011 and 2021. Approximately 1 in 5 women had a low AMH result, according to the fixed or age‐specific reference range documented by the pathology provider. Higher socioeconomic status, not having a healthcare concession card, being a non‐smoker, and residing in a major city were all strongly associated with an increased likelihood of AMH testing. Substantial patient‐ and practice‐ level variation in AMH testing was evident, as was variation in how tests results were reported and flagged as being ‘abnormal’.

### 4.2 Interpretation

To the best of our knowledge, this is the first study to examine the prevalence of AMH testing for fertility assessment. A small cross‐sectional survey of 1773 Australian females aged 18–55 years in 2022 identified that 7% have had an AMH test [12], with 46% of those who received their test through their GP having it undertaken for assessing fertility. The observed rapidly increasing rates of AMH testing in general practice may be driven by broader demographic and commercial trends, including delayed childbearing, greater public awareness of reproductive ageing, increased interest in elective egg freezing, as well as increasing social media targeting of young women to undertake egg freezing and widespread misinformation about the clinical utilities of AMH testing [20, 21]. A content analysis of 27 websites from various countries selling AMH tests direct‐to‐consumers found that most included misleading claims regarding the clinical utility of the AMH test such as its ability to predict fertility potential [22]. Notably, a recent Australian clinical practice survey identified that patients were the ones often initiating the idea of AMH testing, particularly for those who acquired the test via GPs [23].

Our findings highlight significant patient‐ and practice‐level variation in AMH testing. Given the AMH test is not covered by Medicare, Australia's public health insurance scheme, and can cost individuals $100 or more out of pocket [24], it is not surprising that higher socioeconomic status was associated with increased likelihood of AMH testing. The observed high level of variation between general practices in AMH testing rates, independent of differences in patient case mix, is notable and indicates that efforts to improve clinician understanding of the appropriate use of AMH tests may be warranted.

A notable observation was that around one third of AMH test results were reported with a fixed dose reference range, irrespective of age at test. Given age is the strongest factor influencing both AMH levels and chance of conception, the use of fixed reference ranges that don't correct for age at testing leads to a substantial increase in the proportion of test results flagged as being ‘low’, which may lead to difficulties in interpreting results and unnecessary anxiety and concern regarding test results. Further, even where adjusted reference ranges are provided, the current lack of international assay standards and differing assay methodologies makes comparing levels between pathology providers more challenging and also contributes to difficulties in interpreting AMH results. In a recent survey of Australian clinicians, only half felt confident in interpreting and explaining an AMH result to their patients [23]. Further, while based on a small sample size, the finding that approximately one third of AMH levels initially flagged as ‘low’ were considered ‘normal’ on repeat testing further highlights challenges associated with test use and interpretability. This observed variation, and apparent improvement, in AMH levels is consistent with findings from a recent New Zealand study examining changes in AMH levels over time among 1871 patients [25].

### 4.3 Implications for Practice

Practice recommendations are clear in that AMH levels should not be used as a predictor of the likelihood of natural conception [3, 7, 8, 9, 10, 11]. In addition to being a waste of resources and incurring additional costs, unnecessary tests may also result in increased unnecessary medical intervention [20], as well as psychological harms [26]. Further, there is evidence that women who perceive their result as low report greater emotional distress, decisional regret and changes to reproductive decision‐making [27, 28]. Conversely, high AMH levels may provide false reassurance about future fertility, which could lead women to overestimate their chances of conceiving later in life, despite age being the only true predictor of natural conception [29]. A recent randomised controlled trial found that when provided evidence‐based information on AMH testing, many women exhibited less interest in having the test [29]. Clinicians are encouraged to use available resources when discussing the reliability of AMH testing with their patients [30].

### 4.4 Strengths and Limitations

This study has a number of major strengths including the use of a large nationally representative general practice database containing longitudinal individual‐level data on > 1.3 million females and the ability to capture private pathology requests not eligible for government subsidy. The primary study limitation relates to the identification of AMH tests requested for assessing fertility being reliant on the analysis of free text fields. It is possible that general practitioners were ordering AMH tests on behalf of fertility specialists, or to assist in referring patients for management of infertility through ART. Further, we included tests ordered for ‘fertility’ and ‘infertility’ to capture AMH testing requested by GPs for women seeking information about their current or future fertility, or experiencing difficulty conceiving. We considered these indications together because AMH testing is not recommended as a predictor of spontaneous conception or live birth in these settings. An additional limitation is that reasons for AMH test ordering were missing for approximately 1 in 5 tests ordered by GPs, meaning the true prevalence of testing for fertility is likely to have been underestimated. While data are recorded at the individual patient level, patient data is not linked across different general practices, therefore it is possible to double‐count individuals presenting to different general practices. Lastly, MedicineInsight uses a non‐random sampling process to recruit the practices; however, the distribution of included patients closely resembles figures from the last Australian census [13].

## 5 Conclusion

The prevalence of AMH tests requested by GPs in Australia for fertility assessment has increased more than 13‐fold between 2011 and 2021. The marked rise in inappropriate AMH testing by general practitioners, despite increasing evidence of its inability to predict current or future fertility, is concerning and likely harming women through providing false reassurance or unwarranted anxiety. It is also likely to contribute to increased use of unnecessary and costly interventions, including elective egg freezing. These findings highlight the need for further research to better understand the factors influencing AMH testing and support evidence‐based use of AMH testing in clinical practice.

## Funding

This work was supported by Channel 7 Children's Research Foundation Fellowship, CRF‐210323.

## Conflicts of Interest

J. B. declares receiving royalties from Elsevier as a book editor. All other authors declare no conflicts of interest.

## Supporting information


**Figure S1:** Study cohort selection flow diagram.
**Table S1:** Identification of AMH test reasons.
**Table S2:** Patient characteristics according to the number of Anti‐Mullerian Hormone (AMH) tests requested for fertility assessment among females aged 20–44 years who attended Australian general practice, 2011–2021.

## Data Availability

Data may be obtained from a third party and are not publicly available. The authors do not have permission to share the data as the original dataset belongs to a third party. Requests to access data can be submitted to the data custodians, the Australian Commission on Safety and Quality in Health Care: medicineinsight@safetyandquality.gov.au.
