# Supplementary material for: Trends in Anti‐Müllerian Hormone (AMH) Testing for Fertility Assessment in Australian General Practice, 2011–2021: A Retrospective Open Cohort Study
Source: Aust N Z J Obstet Gynaecol. 2026 Aug 31;66(5):e70179. doi: 10.1111/ajo.70179 (PMC13530425; doi:10.1111/ajo.70179)
Supplement: Supplementary file 1 [file AJO-66-0-s001.docx]

# **Supplemental Table 1.** Identification of AMH test reasons

| **Category** | **Description** | **Free text search terms** |
| --- | --- | --- |
| Fertility/Infertility | Tests where indication related to fertility assessment | Key terms: "SUBFERTILITY", "INFERTILITY", “SECONDARY INFERTILITY”, “TRYING TO CONCEIVE”, “FERTILITY ISSUES”, “DIFFICULTY CONCEIVING”, “FERTILITY CONCERNS”, “TTC”, "DIFFICULTY CONCEIVING", "FERTILITY ISSUES", "IMPAIRED FERTILITY", "FERTILITY - IMPAIRED", "DELAYED FERTILITY", "FERTILITY PROBLEM", “INFERTILITY WORK UP”, “TRYING FOR PREGNANCY”, “FERTILITY CHECK”, “FERTILITY ASSESSMENT”, “CHECK UP”, “FERTILITY”, “?OVARIAN RESERVE”, “PREGNANCY PLANNING”,  “FERTILITY WORK UP”, “FERTILITY TEST”, “FERTILITY INVESTIGATIONS”, “PRIMARY INFERTILITY”, “FERTILITY TESTING”, “FERTILITY SCREEN”, “CHECK FERTILITY”, “PLANNING PREGNANCY”, “PRECONCEPTION”, “FERTILITY PLANNING”, “PRE-PREGNANCY”, “EGG TIMER”, “OVARIAN RESERVE”, “CHECK FERTILITY”, "CHECK AMH", "MONITORING", "HEALTH CHECK", "SCREEN", "ROUTINE CHECK", "PATIENT REQUEST", "GENERAL CHECK", "CHECK UP", "REQUESTING TEST", "REQUESTS AMH", “STARTING FAMILY”, “FAMILY PLANNING”  Spelling variations: "INFITLI", "INFITIL", "PRE-CONSEPTIONAL", "FERITL", "FERIL", "FETIL", "FETRIIT", "GERTILITY", "PRE CONCETPURAL", "PRE COCNEPTION", "PRE COCNEPTION", "PRE CONCEPTION", "FERTL", "FERIT", "PRE CONCP", "PRE ONCEP", "PRE PRGN", "PRECEONCEP", "PREPREG"  Excluded terms: “EGG DONOR”, “CRYOPRESERVATION”, “IVF”, “ICSI”, “EGG FREEZING” |
| PMOS | Tests where PMOS was specified or for symptoms commonly associated with PMOS | Key terms: "PCOS", "POLYCYSTIC", "HIRSUITISM", “HIRSUTISM", "ACNE", "HAIR LOSS", "HAIRLOSS", "HAIR FALL"  Spelling variations: "PCOD" "POCS", |
| Other/  Unknown | All other remaining terms or where there was no free text entry | N/A |

**Supplemental Figure 1. Study cohort selection flow diagram**

Active patients (>2 encounters), 14-44 years old, attending general practice between 2011-2021 (MedicineInsight data)

n=1,397,370

AMH test results available

(n=5,659)

AMH test requested by General Practitioner for fertility assessment (n=8 621)

Excluded: AMH requested test reason PMOS (n=314) or Unknown/other (n=3,125)

AMH test requested by General Practitioner

(n=12,060)

No AMH test requested by General Practitioner

(n=1,339,838)

Excluded: Females with diagnosis of PMOS (n=45,472)

N=1,351,898

# **Supplemental Table 2.** Patient characteristics according to the number of Anti-Mullerian Hormone (AMH) tests requested for fertility assessment among females aged 20-44 years who attended Australian general practice, 2011-2021

| **Variables** | **One AMH Test** | **Two or more AMH Tests** | **RR (95% CI)** |
| --- | --- | --- | --- |
| **Total number of patients** | 8285 | 336 |  |
| **Age at first test years** |  |  |  |
| 20-24 | 247 (3.1%) | 13 (3.9%) | 1.29 (0.74-2.27) |
| 25-29 | 1122 (14.1%) | 34 (10.2%) | 0.76 (0.52-1.11) |
| 30-34 | 2607 (32.8%) | 105 (31.4%) | Reference |
| 35-39 | 2661 (33.5%) | 134 (40.1%) | 1.24 (0.96-1.59) |
| 40-44 | 1302 (16.4%) | 48 (14.4%) | 0.92 (0.66-1.28) |
| **Socioeconomic status** |  |  |  |
| Very Low | 640 (7.8%) | 18 (5.4%) | 0.66 (0.41-1.08) |
| Low | 1094 (13.3%) | 46 (13.9%) | 0.98 (0.71-1.36) |
| Middle | 1489 (18.1%) | 60 (18.1%) | 0.94 (0.70-1.27) |
| High | 1993 (24.2%) | 78 (23.5%) | 0.92 (0.70-1.21) |
| Very High | 3030 (36.7%) | 130 (39.2%) | Reference |
| **Remoteness** |  |  |  |
| Major City | 6311 (76.5%) | 262 (78.9%) | Reference |
| Inner Regional | 1331 (16.1%) | 50 (15.1%) | 0.91 (0.68-1.22) |
| Outer Regional | 495 (6.0%) | 18 (5.4%) | 0.88 (0.55-1.41) |
| Remote & Very remote | 109 (1.3%) | 2 (0.6%) | 0.45 (0.11-1.79) |
| **Health care concession card status** |  |  |  |
| Concession | 7526 (90.8%) | 306 (91.1%) | 0.97 (0.67-1.41) |
| No Concession/Unknown | 759 (9.2%) | 30 (8.9%) | Reference |
| **Smoking status** |  |  |  |
| Smoker | 437 (5.8%) | 14 (4.5%) | 0.78 (0.46-1.33) |
| Ex-Smoker | 2763 (36.7%) | 120 (38.2%) | 1.04 (0.83-1.31) |
| Non-Smoker | 4331 (57.5%) | 180 (57.3%) | Reference |

Abbreviations: AMH, Anti-mullerian hormone; RR, relative risk
